# The Determinants of young Adult Social well-being and Health (DASH) study: diversity, psychosocial determinants and health

**DOI:** 10.1007/s00127-015-1047-9

**Published:** 2015-04-11

**Authors:** Seeromanie Harding, Ursula M. Read, Oarabile R. Molaodi, Aidan Cassidy, Maria J. Maynard, Erik Lenguerrand, Thomas Astell-Burt, Alison Teyhan, Melissa Whitrow, Zinat E. Enayat

**Affiliations:** 1 Kings College London, London, UK; 2 Social and Public Health Sciences Unit, Institute of Health and Wellbeing, University of Glasgow, Glasgow, UK; 3 Leeds Beckett University, Leeds, UK; 4 University of Bristol, Bristol, UK; 5 School of Science and Health, University of Western Sydney, Parramatta, Australia; 6 University of Adelaide, Adelaide, Australia

**Keywords:** Longitudinal study, Ethnicity, Physical and mental health, Psychosocial determinants, Adolescence

## Abstract

**Purpose:**

The Determinants of young Adult Social well-being and Health longitudinal study draws on life-course models to understand ethnic differences in health. A key hypothesis relates to the role of psychosocial factors in nurturing the health and well-being of ethnic minorities growing up in the UK. We report the effects of culturally patterned exposures in childhood.

**Methods:**

In 2002/2003, 6643 11–13 year olds in London, ~80 % ethnic minorities, participated in the baseline survey. In 2005/2006, 4782 were followed-up. In 2012–2014, 665 took part in a pilot follow-up aged 21–23 years, including 42 qualitative interviews. Measures of socioeconomic and psychosocial factors and health were collected.

**Results:**

Ethnic minority adolescents reported better mental health than White British, despite more adversity (e.g. economic disadvantage, racism). It is unclear what explains this resilience but findings support a role for cultural factors. Racism was an adverse influence on mental health, while family care and connectedness, religious involvement and ethnic diversity of friendships were protective. While mental health resilience was a feature throughout adolescence, a less positive picture emerged for cardio-respiratory health. Both, mental health and cultural factors played a role. These patterns largely endured in early 20s with family support reducing stressful transitions to adulthood. Education levels, however, signal potential for socio-economic parity across ethnic groups.

## Background

Changing population dynamics and ageing migrant populations pose important challenges for public health. In 2011, there were 48.9 million foreign-born residents in European Union countries, about 10 % of the total population. In the UK, 20 % of the population is from ethnic minority groups, 55 % in London [1]. There are marked ethnic differences in physical and mental health in adulthood which are not well understood, but have important implications for improving the health of the whole population. In Europe, North America and Australia, people of Black African descent, whether born abroad or not, are at higher risk of psychotic illness compared to the majority population [2]. The prevalence of type 2 diabetes shows a four-fold increase in South Asians (higher among Bangladeshis and Pakistanis than Indians) and a three-fold increase in Black Africans and Caribbeans. Risks of stroke are also higher in these groups [3]. Over the last three decades, death rates from hypertension, stroke, and prostate cancer have remained highest in Black Africans and Caribbeans [4], and from coronary events in South Asians [5]. Asthma prevalence and morbidity also varies by ethnic group, being higher among ethnic minority children compared with White British [6]. In older adults, these ethnic differences cannot be wholly accounted for by risk factors measured in middle age. It is clear that ethnic differences in health change over time. Some studies signal the influence of social and environmental factors through convergence towards the health patterns of the host population [7], some show divergence over time [8], whilst others show the emergence of health differences in childhood [9, 10]. Socio-economic structuring of adult morbidity and mortality within ethnic groups is known [11], but does not account for health inequality between groups. Little is known about how socio-economic and psychosocial environments, behaviours and biology in childhood shape later health and well-being among ethnic groups in the UK.

The Determinants of young Adult Social well-being and Health (DASH) longitudinal study provides unique opportunities to better understand the complex interplay of these factors for children growing up with diversity [12]. A recent Medical Research Council review of British cohort studies highlighted the paucity of population cohorts in early adulthood [13], a time when physical and mental resilience is at its peak, yet when early signs of disease appear. There are also few cohort studies with large samples of ethnic minorities. DASH comprises over 6000 people of diverse ethnicities including White British, Indian, Pakistani, Bangladeshi, Black African, Black Caribbean and mixed ethnicity, who are now in their 20s. The study started in 2002, when the cohort was aged 11–13 years and data were collected on socio-economic and psychosocial factors, cardiovascular, respiratory and mental health.

The conceptual underpinning of DASH draws on life-course models [14]. Considerable evidence indicates that experiences early in the life course (such as intra-uterine and childhood development) [15, 16] can have far reaching consequences for health in later life. Subsequent events, such as change in socio-economic circumstances (SEC), may also alter health trajectories initiated in early childhood. In addition, cumulative exposure to adverse effects of deprivation in childhood and adulthood frequently erode health capital. Historical processes also play a key role. Slavery and post-independence poverty may have a continuing legacy via compromised intrauterine and postnatal growth, which may in turn affect, for example, vascular health in later life. Historical processes also shape social patterns of family life (e.g. family separation) with implications for cognitive, behavioural, and emotional development and child mental health [17, 18].

A key hypothesis in DASH relates the role of psychosocial factors in nurturing the health and well-being of ethnic minorities growing up in the UK. We hypothesised that the positive effects of culturally patterned exposures in childhood, family connectedness and religious involvement in particular, will endure through adulthood and buffer the effect of economic adversity on health. The aim of this paper is to give a broad overview of DASH findings with a particular focus on these psychosocial determinants of health in adolescence. We also discuss some of the methodological issues related to engagement of ethnic minorities in a longitudinal study.

## Design and methods

### Study sample

Details of the design of DASH can be found at http://dash.sphsu.mrc.ac.uk and in a published cohort profile [12]. The sample was recruited between 2002 and 2003, from 51 schools in 10 London boroughs. A total of 6643 students, aged 11–13 years, in first and second years of secondary school, took part in the baseline survey. In 2005–2006, 4785 (88 % of children in 49 schools, 72 % of the cohort), aged 14–16 years, took part in the first follow-up. We focus on findings based on follow-up of the entire cohort in adolescence. We also report on the patterns of continuity for key exposures and outcomes based on a pilot follow-up of 665 participants, aged 21–23 years, completed in March 2014. It examined whether participants could be located at the addresses held for them, best methods for data collection, and consent for bio-marker measures and linkage of health and administrative records. The sample consisted of about 100 individuals per ethnic group (White British, Indian, Pakistani or Bangladeshi, Black African, Black Caribbean, other ethnicity), chosen to give a representative spread by gender and SEC across all 10 London boroughs and 51 schools. Forty-two participated in qualitative interviews identified using stratified purposeful sampling to achieve broad representation across a range of socio-demographic criteria (gender, ethnicity, religion, family type, SEC).

### Measures

See Appendix Table 3 for measures in DASH. We take the position that ethnic identity is dynamic, reflecting historic social and cultural traditions but also the influence of current context. Ethnicity in DASH was measured by self-reported ethnicity, checked against reported parental ethnicity and grandparents’ country of birth. For analysis, Bangladeshis and Pakistanis were combined because of relatively small sample sizes. Both groups were distinct from Indians in that they were more economically disadvantaged and predominantly Muslim. Self-complete questionnaires were also used to collect information on religion (affiliation and frequency of attendance at a place of worship), family life (relationship with parents and family connectedness), SEC [parental employment, the Family Affluence Scale (FAS) [19], family type], psychological well-being, and health behaviours (physical activity, smoking, alcohol). The self-report version of the Strengths and Difficulties Questionnaire (SDQ), was used to screen for child mental health problems [20] and a Total Difficulties Score (TDS) was derived. A higher score indicates greater difficulties. The self-report version correlates well with teacher/parent report versions and clinician-rated assessments [21] and has been validated internationally [22]. Perceived parenting style was assessed using the brief form of the parental bonding instrument (PBI) [23] which measures key dimensions of the parent–child relationship. Two variables—‘care’ (i.e. warmth, support and responsiveness) and ‘control’ (i.e. maturity demands, discipline and supervision) were derived. Biological measures (weight, height, blood pressure etc.) were taken by trained nurses and field assistants. Neighbourhood and school level ecological data (see Appendix Table 3) were linked to participants’ records. Qualitative interviews for the pilot study were semi-structured and covered education, social relationships, ethnicity and identity, and transitions to adulthood.

### Analysis

Details of statistical analyses can be found in other DASH papers [10, 24]. Longitudinal data collected at 11–13 and 14–16 years were analysed using gender-specific linear-mixed models to explore the influence of SEC, psychosocial support (e.g. parenting styles, religion and attendance to a place of worship) on mean TDS. Gender-specific logistic multilevel models were used to assess the influence of these factors, and of TDS, on health behaviours (e.g. currently smoking, currently drinking alcohol), categorised as binary responses. Multiple imputation [25] was used to handle missing data on covariates, generally less than 15 % across most variables.

Separate analyses were conducted with the pilot follow-up sample aged 21–23 years. Linear regression models were used to examine ethnic differences in General Health Questionnaire (GHQ-12) scores [26]. We used a combination of stratified models by ethnicity and interaction terms in our regression models to identify potential ethnic specific effects (e.g. for analyses presented here, we tested ethnicity × parental care/parental control/religion/regularity of attendance). Loss of statistical power cannot be ruled out but there were generally few statistically significant interactions. All analyses used STATA 13.

Qualitative interviews were digitally recorded and transcribed. Transcripts were coded using NVivo 10 through iterative comparison of qualitative and quantitative findings. A priori themes were derived from the questionnaires and a grounded theory approach [27] used constant comparison to identify emerging themes.

## Key findings

DASH gives insights into the diversity of contemporary urban life for young people in London. Eighty-six countries of birth, ~100 languages and ~50 religions were reported. 51 % spoke a language other than English at home. While 63 % of ethnic minorities were UK-born, 87 % had at least one foreign-born parent. Religious affiliation was high in all ethnic minority groups. Christianity was the dominant religion for White British (39 %), Black Caribbeans (78 %) and Black Africans (73 %), Hinduism for Indians (59 %), and Islam for Pakistanis/Bangladeshis (96 %). 21 % of Black Africans were Muslims.

Table 1 shows ethnic distributions of measures of structural adversity, psychosocial support and key health behaviours in adolescence. Ethnic minority adolescents remained generally more exposed to structural adversity than White British, including more racism and, apart from Indians, more socio-economic disadvantage. Black Caribbeans and Black Africans were more likely to be in a lone parent household and South Asians least likely. Ethnic minorities also reported more attendance to a place of worship, higher parental control and less parental care [28], and significantly more engagement in family activities (data not shown) [29]. Ethnic diversity in friendships was common across all groups [24]. In relation to health behaviours, ethnic minorities were generally less likely to report tobacco smoking or alcohol consumption, but this increased with age. Questionnaire responses for smoking were validated with salivary cotinine [30]. Overweight and skipping breakfast (a correlate of childhood obesity), however, were more common among some groups, notably Black Caribbean and Black African girls [31].

**Table 1:** The Determinants of young Adult Social well-being and Health (DASH) study: ethnic distribution of measures of structural adversity, psychosocial support, and health behaviours [percentage (95 % confidence intervals)] at age 11–16 years

	White UK	Black Caribbean	Black African	Indian	Pakistani/Bangladeshi	Other
	*N* = 873	*N* = 779	*N* = 892	*N* = 419	*N* = 446	*N* = 1376
Structural adversity						
11–13 years: low family affluence ≤2^a^	27.61 (24.74, 30.67)	35.30 (32.02, 38.73)*	31.95 (28.97, 35.09)	28.16 (24.06, 32.67)	34.08 (29.82, 38.61)	35.25 (32.76, 37.81)*
14–16 years: low family affluence ≤2^a^	27.26 (24.41, 30.32)	37.36 (34.02, 40.81)*	32.51 (29.51, 35.66)*	24.82 (20.91, 29.19)	29.15 (25.11, 33.54)	32.78 (30.34, 35.30)*
11–13 years: lone parent family	23.60 (20.89, 26.53)	47.11 (43.62, 50.63)*	31.39 (28.43, 34.52)*	5.73 (3.87, 8.41)*	10.76 (8.20, 14.00)*	29.87 (27.51, 32.34)*
14–16 years: lone parent family	19.59 (17.09, 22.36)	42.62 (39.18, 46.13)*	32.06 (29.08, 35.20)*	7.88 (5.65, 10.88)*	13.23 (10.38, 16.71)*	28.92 (26.59, 31.38)*
11–13 years: parental unemployment^b^	13.63 (11.51, 16.07)	15.66 (13.27, 18.39)	19.84 (17.35, 22.59)*	13.13 (10.21, 16.72)	30.72 (26.60, 35.16)*	27.54 (25.25, 29.97)*
14–16 years: parental unemployment^b^	6.76 (5.27, 8.63)	8.09 (6.37, 10.22)	15.81 (13.56, 18.35)*	9.55 (7.08, 12.76)*	24.89 (21.09, 29.12)*	3.85 (2.95, 5.01)*
11–13 years: perceived racism^c^	13.40 (11.30, 15.83)	16.05 (13.63, 18.80)	18.50 (16.08, 21.18)*	19.33 (15.82, 23.40)	27.13 (23.20, 31.45)*	19.90 (17.87, 22.09)*
14–16 years: perceived racism^c^	18.56 (16.11, 21.28)	28.11 (25.06, 31.38)*	33.18 (30.17, 36.35)*	31.03 (26.77, 35.63)*	27.80 (23.84, 32.15)*	29.58 (27.22, 32.05)*
11–13 years: living in neighbourhoods in the most deprived quintile^d^	13.29 (11.19, 15.71)	21.44 (18.69, 24.46)*	27.91 (25.07, 30.95)*	4.53 (2.91, 7.00)*	11.66 (8.99, 14.99)	23.69 (21.52, 26.01)*
14–16 years: living in neighbourhoods in the most deprived quintile^d^	12.83 (10.77, 15.22)	21.44 (18.69, 24.46)*	24.78 (22.05, 27.72)*	10.50 (7.90, 13.82)	15.92 (12.81, 19.62)	22.82 (20.68, 25.11)*
11–13 years: living in neighbourhoods with levels of PM2.5 in the highest quintile of PM2.5	13.52 (11.40, 15.95)	17.20 (14.71, 20.02)	26.79 (23.99, 29.80)*	6.21 (4.26, 8.96)*	8.97 (6.64, 12.00)	21.29 (19.21, 23.54)*
14–16 years: living in neighbourhoods with levels of PM2.5 in the highest quintile	13.52 (11.40, 15.95)	19.38 (16.76, 22.31)*	29.04 (26.15, 32.10)*	2.39 (1.29, 4.38)*	6.05 (4.18, 8.69)*	21.44 (19.35, 23.69)*
Psychosocial support						
11–13 years: highest tertile parental care^e^	41.01 (37.79, 44.31)	37.23 (33.90, 40.68)	35.31 (32.24, 38.51)	38.90 (34.34, 43.67)	39.24 (34.80, 43.86)	36.89 (34.38. 39.48)
14–16 years: highest tertile parental care^e^	24.74 (21.99, 27.72)	19.00 (16.39, 21.91)*	19.51 (17.30, 22.24)*	28.88 (24.73, 33.41)	28.25 (24.26, 32.62)	23.76 (21.59, 26.09)
11–13 years: highest tertile parental control^e^	18.10 (15.68, 20.80)	31.84 (28.65, 35.20)*	37.56 (34.43, 40.79)*	35.56 (31.11, 40.27)*	40.13 (35.68, 44.76)*	32.61 (30.18, 35.13)*
14–16 years: highest tertile parental control^e^	20.05 (17.52, 22.84)	34.66 (31.39, 38.08)*	41.59 (38.40, 44.86)*	39.62 (35.04, 44.39)*	41.26 (36.77, 45.89)*	37.57 (35.05, 40.17)*
11–13 years: attendance at a place of worship: ≥1 per week^f^	6.99 (5.47, 8.88)	39.02 (35.65, 42.50)*	65.25 (62.06, 68.30)*	42.96 (38.29, 47.76)*	61.66 (57.05, 66.07)*	27.25 (24.96, 29.67)*
11–13 years: attendance at a place of worship: never attending^f^	57.85 (54.54, 61.09)	13.22 (11.02, 15.79)*	5.38 (4.08, 7.07)*	5.73 (3.87, 8.41)*	9.19 (6.84, 12.25)*	30.67 (28.29, 33.16)*
14–16 years: attendance at a place of worship: ≥1 per week^f^	5.84 (4.47, 7.61)	32.61 (29.40, 35.98)*	67.94 (64.80, 70.92)*	37.47 (32.96, 42.21)*	57.40 (52.75, 61.92)*	25.15 (22.92, 27.51)*
14–16 years: attendance at a place of worship: never attending^f^	67.58 (64.40, 70.61)	18.74 (16.15, 21.64)*	5.61 (4.27, 7.32)*	8.83 (6.46, 11.96)*	13.00 (10.18, 16.46)*	37.43 (34.91, 40.02)*
11–13 years: ethnically diverse friendships^g^	27.38 (24.52, 30.43)	28.37 (25.31, 31.64)	28.48 (25.61, 31.53)	25.30 (21.36, 29.69)	27.80 (23.84, 32.15)	22.53 (20.40, 24.81)
14–16 years: ethnically diverse friendships^g^	33.33 (30.28, 36.53)	30.94 (27.79, 34.28)	35.54 (32.46, 38.74)	40.33 (35.73, 45.11)	42.83 (38.30, 47.47)*	30.01 (27.65, 32.49)
Health behaviours						
11–13 years: currently smoking	3.89 (2.79, 5.40)	3.98 (2.81, 5.60)	0.90 (0.45, 1.78)*	1.19 (0.50, 2.84)	1.79 (0.90, 3.55)	3.56 (2.70, 4.68)
14–16 years: currently smoking	23.37 (20.68, 26.29)	10.78 (8.79, 13.16)*	4.60 (3.40, 6.18)*	5.73 (3.87, 8.41)*	9.64 (7.23, 12.75)*	14.10 (12.36, 16.04)*
11–13 years: currently drinking alcohol	21.42 (18.82, 24.27)	11.94 (9.84, 14.41)*	4.48 (3.31, 6.06)*	1.43 (0.64, 3.15)*	0.45 (0.11, 1.78)*	8.21 (6.87, 9.79)*
14–16 years: currently drinking alcohol	66.90 (63.70, 69.94)	45.31 (41.84, 48.83)*	23.54 (20.87, 26.44)*	20.76 (17.14, 24.92)*	1.35 (0.60, 2.96)*	39.39 (36.84, 42.00)*
11–13 years: skipping breakfast	33.56 (30.50, 36.77)	44.93 (41.46, 48.44)*	48.99 (45.72, 52.27)*	25.78 (21.81, 30.19)*	32.51 (28.32, 37.01)	34.30 (31.84, 36.85)

* *p* < 0.05, significantly different from White UK

^a^Family Affluence Scale derived from number of cars or vans, computers, and holidays [24]

^b^Parental employment adjusted for family type, defined as living with 2 parents and both employed (reference category), one parent and employed, 2 parents and neither employed, one parent and not employed, other family type [51]

^c^Measured by responses to question ‘Has anyone made you feel bad or hassled you because of your race, skin colour or where you were born?’ (at school; at home; on the street or in public) [33]

^d^Income domain of the Index of Multiple Deprivation [35]

^e^Perceived parental care and control measured using the Parental Bonding Instrument [22]

^f^Measured by responses to question ‘How often, outside of school, do you go to a church, other place of worship or for religious education?’ (once a week or more; about once a month; a few times a year; never) collapsed into 3 categories (at least once a week; less than once a week; never)

^g^Measure of cultural integration [39]

Findings from the pilot follow-up at 21–23 years suggest a shift towards socio-economic parity across ethnic groups in relation to higher education. Forty percent of White British had completed a degree, compared with 57 % Indians and Black Africans, 49 % Pakistanis/Bangladeshis, and 34 % Black Caribbeans. Ethnic differences remained, however, in perceived racism [32], parental control, and attendance to a place of worship. Twenty percent of White British, 52 % of Black Africans and 38 % of Indians reported racism. Higher proportions of ethnic minority groups reported high parental control, ranging from 33 % of Black Africans to 7 % of White British. Similarly, attendance at a place of worship at least once a week remained more common for ethnic minorities (Whites 2 %, Indians 23 %, Pakistani/Bangladeshis 39 %, Black Africans 47 %, Black Caribbeans 23 %).

### Better mental health despite structural adversity in adolescence

**Fig. 1:**
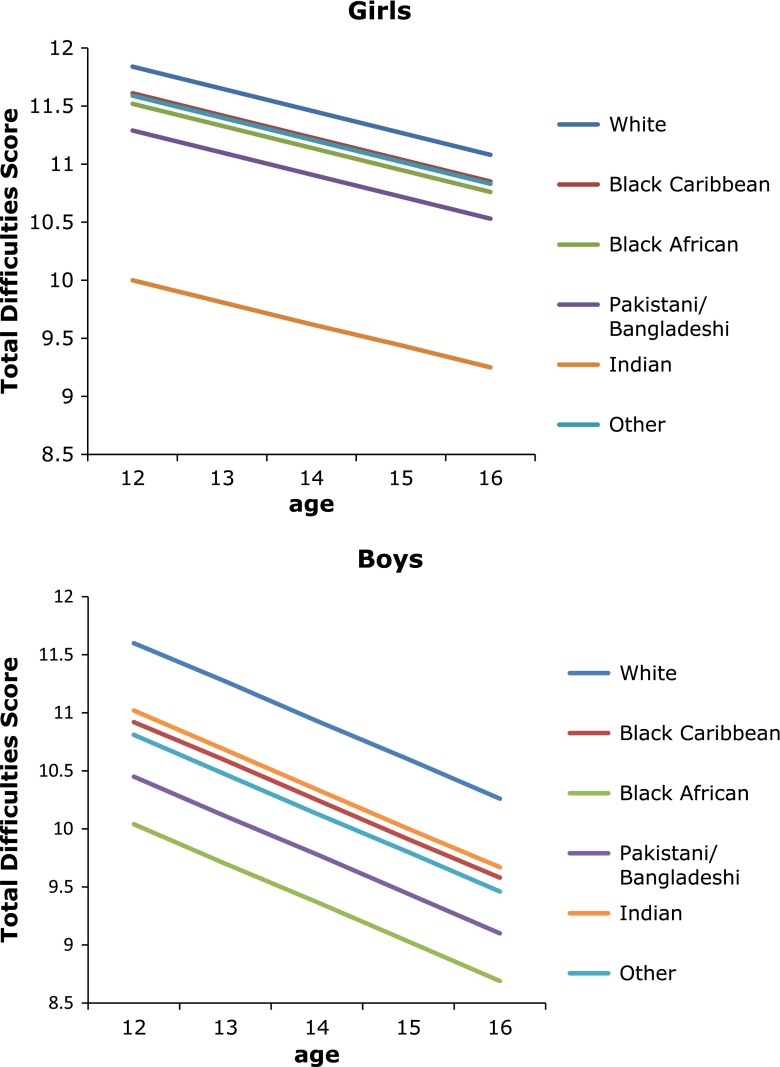
The Determinants of young Adult Social well-being and Health (DASH) study: Mean Total Difficulties Score by age, ethnicity and gender. Total Difficulties Score derived from the Strengths and Difficulties Questionnaire [19]. Score summed from 20 items assessing conduct problems, hyperactivity, emotional symptoms, and peer problems. Age-specific mean scores predicted from gender-specific linear-mixed models adjusted for age, ethnicity, born in the UK or abroad, Family Affluence Scale [24], parental employment, family type, family activities, place of worship attendance, religious affiliation, parental care/control (measured by the Parental Bonding Instrument [22]) × age interaction

Figure 1 illustrates the ethnic patterning of mean TDS. Ethnic minority boys and girls in DASH generally reported better psychological well-being than White British throughout adolescence. Lowest TDS was consistently observed for Black African boys and Indian girls [24, 28, 29, 33]. These patterns remained after adjustment for whether born in the UK or abroad, individual SEC, psychosocial support, and neighbourhood (based on Carstairs index [34]) and school (e.g. percentage eligible for free school meals) SEC. We also examined ‘probable clinical cases’ using a cut-off of TDS >17 (http://www.sdqinfo.org/py/doc/c0.py) by gender and ethnicity, adjusted for potential modifying factors. The pattern by ethnicity was similar to that seen for mean TDS.

In contrast to boys, girls born abroad had better psychological well-being compared with UK-born girls (mean difference in TDS −0.50, 95 % confidence interval −0.95 to −0.05). This effect did not differ by ethnicity. Unlike other measures of adversity, perceived racism was consistently associated with poorer psychological well-being across gender, ethnicity and age. For example, mean difference in TDS between boys who experienced racism and those who did not at age 12 years was −1.88 (−1.75 to −2.01) and at 16 years was −1.19 (−1.07 to −1.31) [35]. Regardless of ethnicity, girls reporting low family affluence had worse psychological well-being which remained significant on adjustment for psychosocial influences (+1.16, 0.21 to 2.10). Living in deprived neighbourhoods was associated with poorer psychological well-being only for White British and Black Caribbeans.

### Parenting, religion, and mental health

**Fig. 2:**
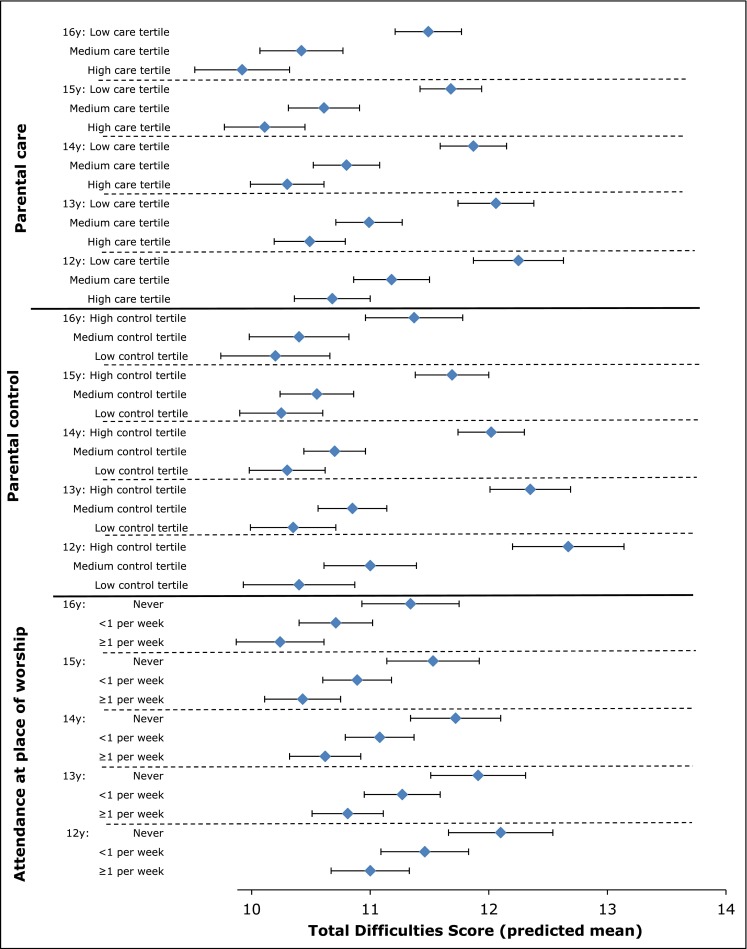
The Determinants of young Adult Social well-being and Health (DASH) study: Girls—Total Difficulties Score by age and, parental style and by age and attendance to a place of worship. Total Difficulties Score derived from the Strengths and Difficulties Questionnaire [19]. Score summed from 20 items assessing conduct problems, hyperactivity, emotional symptoms, and peer problems. Mean score predicted from linear-mixed models adjusted for age, ethnicity, born in the UK or abroad, Family Affluence Scale [24], parental employment, family type, family activities, place of worship attendance, religious affiliation, and parental care/control (measured by Parental Bonding Instrument [22]) × age interaction

**Fig. 3:**
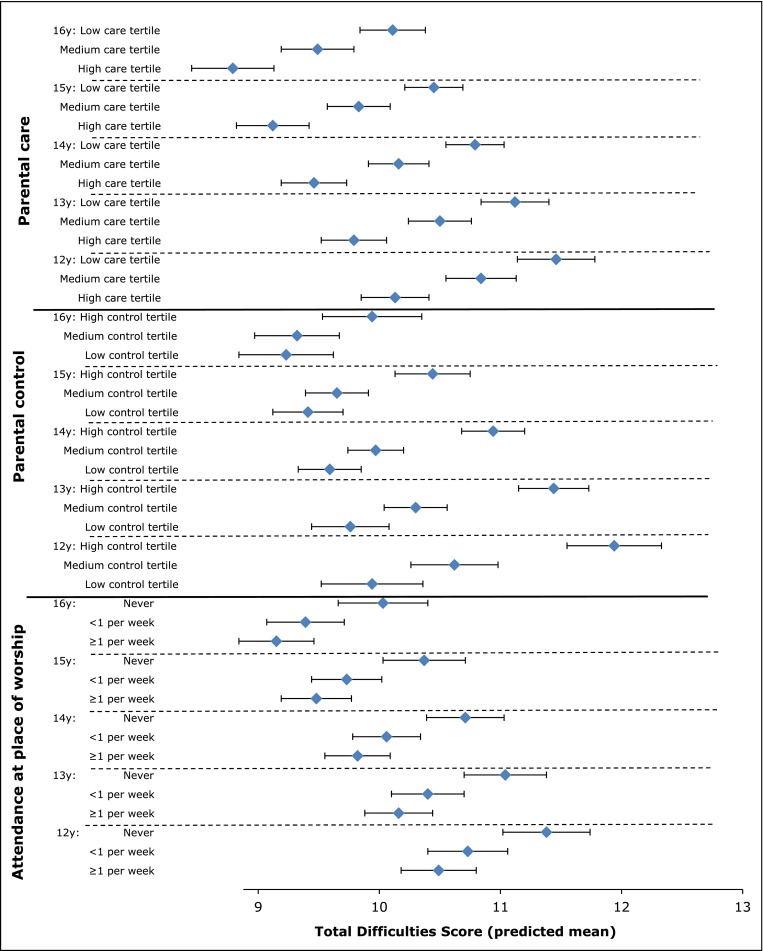
The Determinants of young Adult Social well-being and Health (DASH) study: Boys—Total Difficulties Score by age and parenting style and by age and attendance to a place of worship. Total Difficulties Score derived from the Strengths and Difficulties Questionnaire [19]. Score summed from 20 items assessing conduct problems, hyperactivity, emotional symptoms, and peer problems. Mean score predicted from linear-mixed models adjusted for age, ethnicity, born in the UK or abroad, Family Affluence Scale [24], parental employment, family type, family activities, place of worship attendance, religious affiliation, and parental care/control (measured by Parental Bonding Instrument [22]) × age interaction

Other recent studies report a similar ethnic minority advantage in mental health [36, 37], but it remains unclear what accounts for this apparent resilience. In DASH, regardless of ethnicity, measures of parental care, family connectedness, cultural integration (measured by ethnic diversity of friendships [38]), and frequency of attendance at a place of worship were consistently associated with a protective effect on mental health even after adjustment for SEC [24, 28, 29]. Figures 2 and 3 illustrate this for parenting and place of worship attendance. Increasing parental care, decreasing parental control, and increasing frequency of attendance were independent correlates of better psychological well-being. Religious affiliation itself was not associated with mental health. We also examined the influence of potential psychosocial support from ethnic density and diversity in schools and communities [35]. High own group ethnic density has been hypothesised to be protective of mental health as a result of common social norms and access to support networks [39]. In contrast to studies with adults [40], DASH showed little evidence of an effect of own group ethnic density or diversity on psychological well-being [35].

The interpretation of these findings is complex. The consistency of TDS patterns across ethnic groups argues against any substantial reporting bias by ethnicity. However, cultural influences on family life appear to play an important role. We previously reported that the pattern of relatively better mental health compared with White British was observed among those who reported least autonomy (high parental control) in low SEC as well as two-parent families [28]. Social ecological approaches recognise interactions between individual, social and environmental factors in fostering resilience, including cultural patterns of family life and access to opportunities and resources [41]. The parenting experiences of ethnic minorities are consistent with an ‘authoritative’ parenting style combining warmth and support with a disciplinary framework, considered optimal for child development [42, 43] and academic achievement [44]. Specific goal-directed parenting practices, such as helping with homework or engaging children in family social functions, may also be beneficial [45].

DASH findings are partially consistent with evidence for a protective effect from religious involvement on mental health in adolescence [46, 47]. Religion is thought to influence mental health through social support, social regulation, a sense of meaning and coherence, and positive coping [48]. Regardless of religious affiliation, attendance to a place of worship provides wider family support from a community with shared norms and values [49]. For example, if greater parental control is considered normative in a community, religious involvement may reinforce this parenting style, the children may not perceive the relative lack of autonomy to be unfair, and its influence on mental health may be less negative than for White British children. The qualitative interviews (reported below) gave insight into such intersections between religion, culture and family life.

### Cardio-respiratory health and psychological well-being in adolescence

While mental health resilience among ethnic minorities has been a feature throughout adolescence in DASH, a less positive picture emerged for physical health. In summary, DASH has shown systematic differences in cardio-respiratory health risks in adolescence. For example, at age 12 years, many ethnic minority groups had lower systolic blood pressure (BP) than White British, but greater age-related increases by 16 years. By age 16 years, African boys had significantly higher BP than White British, after accounting for differences in body size [10]. Lower lung function for most minority groups compared with White British persisted after taking into account anthropometric differences [50], and there was more asthma in Black Caribbean boys. As reported above, there were also significant ethnic differences in overweight [51], with skipping breakfast, maternal overweight and maternal smoking key correlates across all groups [51, 52].

**Fig. 4:**
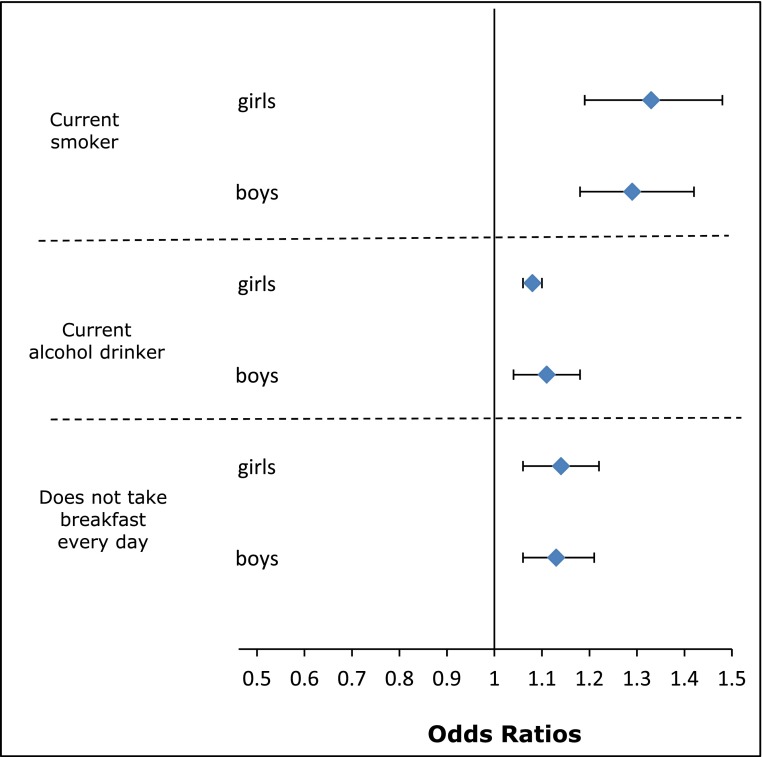
The Determinants of young Adult Social well-being and Health (DASH) study: Association between Total Difficulties Score and health behaviours at age 11–16 years: odds ratio and 95 % confidence interval for smoking, drinking alcohol and skipping breakfast, by gender. Total Difficulties Score derived from the Strengths and Difficulties Questionnaire [19]. Score summed from 20 items assessing conduct problems, hyperactivity, emotional symptoms, and peer problems. Odds ratios derived from logistic multilevel models for smoking and drinking alcohol measured at 11–13 and 14–16 years, and logistic models for skipping breakfast only at 11–13 years. Models adjusted for age, ethnicity, born in the UK or abroad, Family Affluence Scale [24], parental employment, family type, place of worship attendance, religious affiliation, parental care and parental control (measured by the Parental Bonding Instrument [22])

Psychosocial support mattered for some of these outcomes. Table 2 shows that attendance at a place of worship less than once per week and low parental care were both independently related to current tobacco smoking, alcohol use, and skipping breakfast. Ethnic patterns of generally less smoking or alcohol use were maintained after adjusting for SEC, parenting and religious involvement. In contrast to TDS, religious affiliation was independently associated with some behaviours. For example, Hindu girls and Muslim boys and girls were less likely to smoke or drink alcohol, regardless of frequency of attendance to a temple or mosque. There was also some evidence of the varying relationship between ethnicity, religion and place of worship attendance. For example, the lower likelihood of drinking alcohol with increasing frequency of attendance appeared to be more distinct for Pakistani/Bangladeshi or Muslim boys than for White British or Christian boys. Psychological well-being was also associated with some of these outcomes although reverse causality is possible. Adjusted for SEC, poorer psychological well-being was associated with lower lung function [49], and a higher likelihood of asthma [52]. Figure 4 illustrates a similar pattern with increasing mean TDS associated with a greater likelihood of smoking, alcohol consumption, and skipping breakfast.

**Table 2:** The Determinants of young Adult Social well-being and Health (DASH) study: influence of parenting styles and frequency of attendance to a place of worship on health behaviours at age 11–16 years: odds ratios (95 % confidence interval)

	Current smoker	Current alcohol drinker	Does not take breakfast everyday
Girls	*N* = 352	*N* = 965	*N* = 980
Parental care (high care tertile = 1.00)^a^			
Middle tertile parental care	1.34 (0.94, 1.93)	1.74 (1.36, 2.22)*	1.19 (0.95, 1.50)
Low tertile parental care	2.62 (1.84, 3.72)*	2.54 (1.98, 3.26)*	1.96 (1.53, 2.51)*
Parental control (low control tertile = 1.00)^a^			
Middle tertile parental control	0.98 (0.71, 1.35)	0.83 (0.66, 1.04)	0.99 (0.78, 1.26)
High tertile parental control	1.88 (1.35, 2.62)*	0.91 (0.72, 1.16)	0.83 (0.64, 1.08)
Attendance at place of worship (≥1 per week = 1.00)			
<1 per week	2.01 (1.42, 2.85)*	2.10 (1.64, 2.67)*	1.13 (0.89, 1.44)
Never	2.60 (1.72, 3.94)*	2.43 (1.78, 3.32)*	1.57 (1.15, 2.13)*
Boys	*N* = 306	*N* = 920	*N* = 824
Parental care (high care tertile = 1.00)^a^			
Middle tertile parental care	1.22 (0.87, 1.71)	1.26 (1.00, 1.58)	1.42 (1.13, 1.79)*
Low tertile parental care	1.27 (0.89, 1.81)	1.78 (1.42, 2.23)*	1.64 (1.31, 2.06)*
Parental control (low control tertile = 1.00)^a^			
Middle tertile parental control	0.96 (0.70, 1.33)	0.87 (0.71, 1.08)	1.00 (0.79, 1.27)
High tertile parental control	1.37 (0.96, 1.94)	0.96 (0.76, 1.22)	1.13 (0.89, 1.45)
Attendance at place of worship (≥1 per week = 1.00)			
<1 per week	2.27 (1.55, 3.34)*	1.45 (1.13, 1.86)*	0.92 (0.73, 1.16)
Never	2.27 (1.42, 3.62)*	1.55 (1.14, 2.11)*	1.08 (0.80, 1.46)

Odds ratios derived from logistic multilevel models for smoking and alcohol measured at 11–13 and 14–16 years, and logistic models for skipping breakfast only at 11–13 years. Models adjusted for age, ethnicity, where born, Family Affluence Scale [24], parental employment, family type, place of worship attendance, religious affiliation, parental care, and parental control measured by Parental Bonding Instrument [22]

* *p* < 0.05

^a^Perceived parental care and control measured using the Parental Bonding Instrument [22]

### Tracking of resilience at 21–23 years in the pilot follow-up sample?

A key question is whether the relative mental health advantage found in ethnic minority adolescents will erode with age and whether positive psychosocial influences in childhood will continue to have a favourable effect in adulthood. While interpretation of the findings from the pilot follow-up at 21–23 years should be viewed with caution because of the small sample size (*N* = 665), the pattern of GHQ-12 scores among ethnic minority groups indicates potential continuation of the mental health advantage into early adulthood. Adjusted for gender, SEC and psychosocial support, and compared to White British (GHQ mean 12.4), there was a pattern of lower GHQ scores in Black Caribbeans (−1.22, 95 % confidence interval −3.23 to 0.80), Black Africans (−2.56, −4.65 to −0.46), Indians (−3.19, −5.86 to −0.52), and Pakistanis/Bangladeshis (−0.37, −3.03 to 2.29). GHQ scores were higher for females (+2.27, 1.14 to 3.41) than males (GHQ mean 10.2), among those who reported low maternal care (+2.85, 1.33 to 4.36) than high care (GHQ 9.7), and among those who reported high maternal control (+1.90, 0.28 to 3.53) than low control (GHQ 10.5). In contrast to what was observed in adolescence, religion or place of worship attendance did not appear to be associated with mental health. Small sample sizes prevent reliable inferences, but the continuity and discontinuity of these patterns in adulthood raise intriguing questions as to why parenting, but not attendance to a place of worship, remained significant. Arguably whilst the experience of parenting may have remained similar for participants in their 20s, what it means to attend a place of worship may have changed.

### Perceptions of childhood experiences and transition to adulthood

Qualitative interviews at 21–23 years enhanced the interpretation of quantitative findings on mental health in adolescence and young adulthood. In a separate paper, we report on the cultural appropriateness of the GHQ comparing data from questionnaire responses and qualitative interviews. In brief, the GHQ-12 appears to be conceptually congruent regardless of ethnicity; however, known sensitivity to temporal effects may have had some effect on mean scores. High mean scores in the pilot study sample may be a consequence of acute stresses related to life-stage transitions particularly study, financial difficulties or finding work. One participant who scored 7 on the GHQ bimodal score (indicating poor psychological well-being) stated: “Lately it’s because like I haven’t been able to find a job. So I feel like, ‘Oh my God, I don’t have any money, how am I going to live?’ Stuff like that. That’s what’s really straining me right now” (Participant 25, Female, Bangladeshi, degree, one parent).

Key themes emerged in the qualitative analysis around the influence of family life and religion on well-being through a variety of intersecting, mutually reinforcing effects including: group membership and cultural identity, social and instrumental support, moral guidance, aspirations and sense of purpose, and coping strategies (Box 1). These map onto factors which have been identified as fostering resilience from childhood into early adult life including identity formation, social support, ‘planfulness’ (future motivation) and coping skills [53].

Continued commitment to family life and values from childhood into adulthood was a recurring theme regardless of family type. Several participants described the absence of a father from the household due to separation, divorce or migration, however, access to networks of support through extended and transnational family ties was common. Most participants were living in the family home at 21–23 years facilitating the continuation of close family ties and access to social and instrumental support during study or insecure, low-paid employment. Higher reported parental control among ethnic minorities may reflect cultural and religious influences on parenting styles with “strict”, “traditional” or “authoritative” parenting based on respect for elders and those in authority linked to perceived “Asian”, “Caribbean” or “African” cultural values, as well as Christian or Islamic teaching.

Many described such parenting as shaping aspirations in adult life. In participants’ accounts parental discipline was often combined with encouragement to “work hard” and “do your best”. Ethnic minority participants expressed high educational and career goals nurtured by parents’ aspirations and an awareness of opportunities for social mobility resulting from parents’ migration and hard work, often in low-paid, low-status jobs such as cleaners, security guards and taxi drivers. The ethnic parity in higher education status at 21–23 years in DASH is comparable to that reported for 16–24 years olds in the 2011 census and a striking contrast to ethnic differences in education status for their parents’ generation [54]. Selection factors might also have played a role. As migrants, their parents are likely to have experienced downward mobility on migration and fewer opportunities for upward mobility [55]. In contrast, the White British population may have lived in deprived London areas for several generations and partly represent a residual population from which the more aspiring have left. Though aspirations generally appeared to enhance well-being among DASH participants, for some they were experienced as stressful, placing them under “pressure” to achieve.

Religious practice and the instillation of religious values were an important part of family life and cultural identity in childhood for many ethnic minority participants. For Muslim and Hindu participants, this included explicit prohibition on the use of drugs and alcohol but family and cultural values of self-discipline, forbearance and altruism were described across religious and ethnic groups. Places of worship or religious societies were reported to provide social and instrumental support, for example providing friendship networks when moving to university outside London or help finding work. Religious practices and values were described as “our culture” forming an important part of processes of cultural socialisation and solidarity. This was supported by shared religious orientations with friends and peers as well as family. Religion was discussed as providing a sense of meaning and purpose in life in the face of adversity, including experiences of discrimination and racism. Though religious practice was discontinued by some in adulthood, for African Christian and Pakistani/Bangladeshi Muslim participants in particular, religion remained an important influence on identity, lifestyle, aspirations and values, as well as providing coping strategies for life challenges such as praying for guidance, retaining a sense of self-worth, and keeping in mind “the bigger picture”.

### Potential for follow-up

Around 80 % of the cohort has been located, now aged 21–23 years, and 665 participants (90 % of those invited) took part in the feasibility study. Flexibility in interview locations and appointment times boosted participation rates, with one-third seen at weekends or in the evening. Interview locations offered included General Practitioner surgeries and community pharmacies within 2 km of residential postcodes. Overall, regardless of ethnicity or interview location, response rates to all measures and quality of data collected were high (less than 10 % missing data in any interview setting). For all ethnicities, consent rates ranged between 85 and 90 % for new biomedical measures and linkage of health and administrative records. There is strong potential for a three-generation study with consent for parents of the cohort to be contacted and about 300 babies born to participants. Feasibility study findings will be reported fully in subsequent papers.

### Strengths and limitations of DASH

In 2002/2003, empirical studies of ethnicity and health on such a large scale were unprecedented in the UK. The cohort is well characterised in relation to socio-economic, psychosocial, physical and mental health measures. The qualitative study at 21–23 years has provided nuanced exploration of contextual influences on health and well-being. Attrition has been kept low by regular contact with cohort members and their communities, consultation with a participant advisory group, and use of social media and electronic tracing. Multidisciplinary input from social and biomedical scientists provides a platform for general population health studies and for specialist sub-studies. Lessons learnt about successful methods of engaging populations conventionally thought ‘hard to reach’ have been valuable to the science of maintaining representativeness of cohorts.

DASH findings have also informed follow-on studies including intervention studies using participatory approaches to optimise sustainability of behaviour change, including PROmoting Wellbeing, Equality, and Support in breast cancer Survivorship (PROWESS) (http://controlled-trials.com/isrctn/pf/14016157), the Size and Lung function in Children (SLIC) study [56], and the DiEt and Active Living (DEAL) study [57]. Key measures of exposures and outcomes in DASH are harmonised with those in other cohorts giving scope for comparative studies. Planned linkage of primary and secondary health records will capture some early life exposures (such as birth details for those born in the UK), and also pregnancies, births and later health (e.g. admissions to hospital, cancer or death registrations). Cohort members are based in London which is unique in the extent of social and cultural diversity. Nonetheless, increasing globalisation and migration mean that the effects of urban context, deprivation, culture and ethnicity on health and well-being carry implications for other urban settings in the UK and abroad.

In summary, the DASH study focuses on a generation of ethnically diverse children who were born in the 1990s and have grown up in London. It provides a unique opportunity to examine how biology, behaviours and socio-economic and psychosocial environments in childhood shape health and well-being in adulthood. There are no other contemporary British-based cohorts that can be used to examine the role of childhood circumstances in ethnic-gender transitions to adulthood in times of economic precariousness. As expected from national statistics [58], young adults in DASH faced stressful transitions to adulthood with low-paid, often part-time, insecure employment. Although there appeared to be parity by educational level across ethnic groups, increases in racism [59] may frustrate aspirations.

DASH points to the importance of adherence to traditions of family life and religion, and of cultural adaptability, such as living with diversity, in nurturing mental health and well-being [41]. In contrast, greater cardiovascular risks in ethnic minority adolescents indicate a continuation of the biological legacy of earlier generations. A key question is whether culturally based psychosocial experiences in childhood that appear to have fostered psychological resilience will also mitigate biological ‘wear and tear’ and reduce the high cardiovascular disease risks of their parents’ generation. The successful pilot follow-up opens up unique opportunities to track the health and well-being of young Londoners. Researchers can access information about the DASH study and scientific publications through the study website, dash.sphsu.mrc.ac.uk, where data information, data sharing policies and application forms for data access are available, and also via https://www.datagateway.mrc.ac.uk/search/site/DASH.

## Appendix

See Table 3.

**Table 3:** The Determinants of young Adult Social well-being and Health (DASH) study: summary of data collected

	11–13 years (*N* = 6634)	14–16 years (*N* = 4779)	21–23 years (feasibility sample *N* = 665)
Physical measures by trained field assistants/nurses			
Standing height	X	x	x
Sitting height	X	x	x
Weight	X	x	x
Waist circumference	X	x	x
Hip circumference	X	x	x
Pubertal stage	X	x	
Arm circumference	X	x	x
Blood pressure	X	x	x
Jugulum-symphysis distance^a^			x
Pulse wave velocity, augmentation index (TensioWin Arteriograph 24)			x
Bio-impedance		x	x
Lung function (FEV, FVC, PF)	X		x
Cotinine (measured from saliva sample)	X		
25 ml blood sample (biochemistry, haematology, gut hormones, extraction of DNA, storage)			x
Accelerometry data (activPAL)			x
Self-complete questionnaires			
Physical activity diary			x
24 h recall dietary diary			x
Main questionnaire: socio-economic circumstances, family life, psychosocial measures, lifestyle, health, parental health	X	x	x
Ecological data linked at LSOA level			
Pollution	X	x	
Green space	X	x	
Crime rates	X	x	
Deprivation	X	x	
Ethnic density	X	x	
Ecological data linked at school level			
School performance	X	x	
Absenteeism and truancy	X	x	
Free school meals	X	x	
Qualitative interviews			
Topics: employment/study, finances, housing, ethnic and religious identity, relationships with family/peers, physical and mental health, opportunities and challenges in transition to adulthood, understanding the General Health Questionnaire			x

^a^Jugulum-symphysis distance is the distance between the participant’s jugulum and the symphysis and is used to calculate the augmentation index and pulse wave velocity using the Arteriograph 24

## Box 1: the Determinants of young Adult Social well-being and Health (DASH) study: qualitative interview excerpts at age 21–23 years

*Group membership and cultural identity*

…growing up in a Nigerian house and also knowing the British way of life, the difference in respect of your elders and your parents are quite different. […] with Nigerians a child has to respect an elder and that is the key to almost everything

(Participant 42, male, Black African, Christian, degree, two-parent)

I think, religion, culture, especially amongst, like, Muslims, or if you want to say Pakistani people, it tends to be close as well, so you will be close to your family.

(Participant 12, male, Pakistani, degree, two-parent)

*Social and instrumental support*

At university it helped a lot, I was part of the Hindu society so that helped a lot. Like, just meeting new people, communicating with people, like businesses and stuff like that

(Participant 17, male, Indian, Hindu, degree, two-parent)

I’ve always from day dot had my father’s support, I think that’s why, you know—the thing is I never have to worry in my life because I’ve got my dad in my life.

(Participant 11, male, Pakistani, Muslim, GCSE^a^, two-parent)

*Moral guidance*

What our religion’s based on is just, like, it’s like living your life in the most gracious way, being healthy, it’s not killing yourself with, you know, smoking and all the bad things for you

(Participant 22, female, Pakistani, Muslim, GCSE, lone parent)

…it’s influenced me in a positive way because I feel like being brought up that way has made me who I am and made me go the right path, rather than going the wrong path

(Participant 21, female, Black African, Christian, degree, two-parent)

*Aspirations and sense of purpose*

I want to buy a house, I want to buy that by next year, so I mean, I’m putting down, I’m not going shopping, I’m not spending on alcohol, I’m not spending on drink or cigarettes or anything, I’m not, you know, doing anything I shouldn’t be doing, I’m putting it down for my house.

(Participant 29, female, Black Caribbean, Christian, degree, lone parent)

…it’s probably a mixture of religion, my upbringing and my own personality. I think when you grow up, when you’re told, “This is who you are”, or I guess like, God-wise, it’d be like, “This is why you’re created”, or “There is purpose” […] I’ve always grown up with that kind of sense.

(Participant 30, female, Black African, Christian, degree, lone parent)

*Coping strategies*

I might be in a situation where I’m like, “Oh, I don’t know what to do.” So I’ll pray about that. And even in times when I’m stressed, for example, when I was at university, I’d pray about that. If I had my exam I’d pray about that because I was so nervous about it.

(Participant 35, female, Black African, Christian, degree, lone parent)

…it helps when you’ve got like, when there’s, like, a bad situation where you’re just going through a really tough time, having something to, kind of, to cling to and to, kind of, focus on and understand that obviously bad things happen but, you know, it’s not the end.

(Participant 28, female, Black African, Christian, degree, lone parent)

^a^GCSEs (General Certificate of Secondary Education) are academic qualifications usually awarded on leaving secondary school at age 15–16 years, dependent on successful completion of examinations
